# Special Issue on “Plant Biology and Biotechnology: Focus on Genomics and Bioinformatics 2.0”

**DOI:** 10.3390/ijms242417588

**Published:** 2023-12-18

**Authors:** Yuriy L. Orlov, Ming Chen

**Affiliations:** 1 The Digital Health Institute, I.M. Sechenov First Moscow State Medical University of the Ministry of Health of the Russian Federation (Sechenov University), 119991 Moscow, Russia; 2 Institute of Cytology and Genetics, Siberian Branch of the Russian Academy of Sciences, 630090 Novosibirsk, Russia; 3 Agrarian and Technological Institute, Peoples’ Friendship University of Russia, 117198 Moscow, Russia; 4 Department of Bioinformatics, College of Life Sciences, Zhejiang University, Hangzhou 310058, China

The analysis of molecular mechanisms underlying plant adaptation to environmental changes and stress response is crucial for plant biotechnology. The key approaches include bioinformatics methods, high-throughput sequencing, and post-genome technologies. Sequencing and system biology methods offer a comprehensive view of plant growth from molecular to cellular, organ, and population levels. Genomics and bioinformatics facilitate the modeling of protein–protein and gene regulatory interactions in plant cells, providing a basis for better crop production and sustainability. Plant–pathogen interaction studies complement network modeling in this area. Considering research topics of interest in the scientific community, we have organized this Special Issue entitled “Plant Biology and Biotechnology: Focus on Genomics and Bioinformatics 2.0” (https://www.mdpi.com/journal/ijms/special_issues/PlantBi_Biology, accessed on 1 December 2023) continuing a previous thematic Special Issue entitled “Plant Biology and Biotechnology” 2022 [1] (https://www.mdpi.com/journal/ijms/special_issues/Plant_Biotechnology, accessed on 1 December 2023).

This Special Issue gathers a series of research topics on bioinformatics, gene expression regulation, and sequencing analysis presented in a previous issue of MDPI’s *International Journal of Molecular Sciences* entitled “Bioinformatics of Gene Regulations and Structure” (https://www.mdpi.com/journal/ijms/special_issues/Bioinformatics_Genomics (accessed on 1 December 2023) and related journal issues published after the series of bioinformatics conferences that were held in Russia—BGRS (https://bgrssb.icgbio.ru/2022/, accessed on 1 December 2023). Recent journal issues on bioinformatics of gene expression include the *International Journal of Molecular Sciences* (https://www.mdpi.com/journal/ijms/special_issues/0LGA6103S5, accessed on 1 December 2023); *Life* (https://www.mdpi.com/journal/life/special_issues/computational_genomics_life, accessed on 1 December 2023) [2]; *Frontiers in Plant Sciences* (https://www.frontiersin.org/research-topics/54136/applications-of-artificial-intelligence-machine-learning-and-deep-learning-in-plant-breeding, accessed on 1 December 2023); and the *Journal of Integrative Bioinformatics* [3]. The PlantGen2023 conference on plant genetics, genomics, bioinformatics, and biotechnology, held in Kazan City in Russia in 2023, raised several points of discussion regarding biotechnology methods [4] (https://plantgen2023.ofr.su/, accessed on 1 December 2023). Based on the presentations at these conferences, we review the current trends in plant bioinformatics oriented toward transcriptome sequencing data analysis and applications of machine learning methods.

In this Special Issue, we have amassed studies that focus on gene expression regulation in plants and the underlying molecular mechanisms of plant development and stress response using new bioinformatics tools. This Special Issue contains eight research manuscripts each concerning a bioinformatics solution or genomic application to a given plant model (Contributions 1–8).

We start this issue with research on the molecular mechanisms of DNA binding for the regulation of gene expression by Yan et al. (Contribution 1). Bowei Yan and colleagues (https://doi.org/10.3390/ijms24044078) analyzed gene expression under cold treatment in hemp (*Cannabis sativa* L.) for the members of the diacylglycerol acyltransferase enzyme family that play critical roles in catalyzing triacylglycerol biosynthesis in plants. Cannabis sativa diacylglycerol acyltransferase enzyme genes were upregulated in response to cold stress [5]. Further studies on this gene family will help improve hempseed oil composition.

Serafima Novikova et al. (https://doi.org/10.3390/ijms24054530) studied the genetic adaptation of forest trees to high altitudes (Contribution 2). Siberian larch (*Larix sibirica* Ledeb.) was used as the research object [6]. This work builds on other studies on the genetic and epigenetic mechanisms of longevity in forest trees [7,8]. Single-nucleotide polymorphisms related to the adaptation of Siberian larch were found in the genes involved in the processes of macromolecular cell metabolism and organic biosynthesis, as well as organisms’ stress response.

Yuexia Wang and colleagues (https://doi.org/10.3390/ijms24065226) studied drought stress response in wheat using a transgenic Arabidopsis model (Contribution 3). Psb28, a soluble protein in the photosystem II complex, was earlier defined as one of the most important stress response genes because its upregulation was most likely to be associated with the alleviation of damage to plant chloroplast ultrastructure and photosynthesis. It was found that the overexpression of transgenic Psb28 from wheat (*Triticum aestivum* L.) in Arabidopsis exerts a positive role in the drought response by influencing the functional metabolism of endogenous hormones. Bioinformatics approaches for analyzing plants’ response to adverse environmental factors were discussed in previous special journal issues on plant bioinformatics [1,9] using meta-analyses of transcriptomics data.

Wen Duan et al. (https://doi.org/10.3390/ijms24087189) analyzed the expression patterns of members belonging to the dirigent (DIR) gene family in rice (the genus Oryza) (Contribution 4). Accumulating evidence suggests that the expression of many DIR genes is strongly affected by various abiotic stimuli [10]. RNA sequencing and PCR assays confirmed the responsiveness of these genes to the insufficient supply of mineral elements, the excess of heavy metals, and plant infection.

The three subsequent studies (Contributions 5, 6, and 7) presented more specific plant models such as Tartary buckwheat, mulberry, and poplar. Huiling Yan et al. (https://doi.org/10.3390/ijms24098090) studied light response in Tartary buckwheat (Polygonales Polygonaceae *Fagopyrum tataricum* (L.) Gaertn) based on histone deacetylase expression (Contribution 5). Tartary buckwheat is a novel plant model for transcriptome studies [11,12]. They found divergent alterations in histone deacetylase transcript abundance in response to different light conditions according to RNA-seq and RT-qPCR data.

Xin Jin et al. (https://doi.org/10.3390/ijms24119650) studied the effects of magnesium nutrient application in mulberry (*Morus alba*) plants (Contribution 6). Mulberry (*Morus* spp.) is cultivated primarily for its leaves and fruits. Mulberry leaves are the only food source for domestic silkworms (*Bombyx mori L*.); thus, mulberry is an indispensable tree species for the sericulture sector [13]. Mulberry growth and development are affected by nutrient levels, especially that of magnesium. Using metabolomics methods, Jin et al. revealed that an adequate supply of Mg promoted physiological response parameters in mulberry, including net photosynthesis, chlorophyll content, leaf and root Mg content, and biomass (Contribution 6).

Yilian Zhao et al. (https://doi.org/10.3390/ijms24044078) applied cytogenetics methods to karyotype construction in poplar (*Populus simonii*) (Contribution 7). The fluorescence in situ hybridization (FISH) results revealed some errors in the current *P. simonii* genome assembly [14]. It was shown that the method known as oligo-FISH, in which pachytene chromosomes are used, is a powerful tool for constructing high-resolution karyotypes and improving the quality of genome assembly.

Lastly, Lidiia Samarina et al. (https://doi.org/10.3390/ijms241914538) investigated nucleotide polymorphism in the tea plant (*Camellia sinensis*) in terms of leaf quality and production (Contribution 8). The purpose of their study was to analyze the leaf quality and SNPs in a series of 20 mutant genotypes in tea grown without nitrogen fertilizers. Significant positive correlations were found between the nitrogen content and biochemical parameters, such as theanine, caffeine, and most catechins. This contribution continued previous studies on the bioinformatics of plant stress response [15,16].

Thus, a variety of plant species [17] have to be studied through sequencing analysis methods to explore gene expression regulation [17,18]. Machine learning models and artificial intelligence applications present new trends in plant science [19,20,21]. The problems associated with plant bioinformatics and discovering the molecular mechanisms of gene expression in plant models are presented in several Special Issues devoted to the works on gene expression regulation, including Special Issues in *Frontiers in Genetics* [22,23] (https://www.frontiersin.org/research-topics/8383/bioinformatics-of-genome-regulation-and-systems-biology) and *Frontiers in Plant Science* [20,21] (https://www.frontiersin.org/research-topics/54136/applications-of-artificial-intelligence-machine-learning-and-deep-learning-in-plant-breeding, accessed on 1 December 2023), and a Special Issue entitled “New Sights into Bioinformatics of Gene Regulations and Structure”, presented in the *International Journal of Molecular Sciences* (https://www.mdpi.com/journal/ijms/special_issues/MVA479KFR7, accessed on 1 December 2023). We hope that readers find these contributions interesting and stimulating, and we will continue to gather novel research on bioinformatics for ongoing topical issues.
